# MR radiomics predicts pathological complete response of esophageal squamous cell carcinoma after neoadjuvant chemoradiotherapy: a multicenter study

**DOI:** 10.1186/s40644-024-00659-x

**Published:** 2024-01-23

**Authors:** Yunsong Liu, Yi Wang, Xin Wang, Liyan Xue, Huan Zhang, Zeliang Ma, Heping Deng, Zhaoyang Yang, Xujie Sun, Yu Men, Feng Ye, Kuo Men, Jianjun Qin, Nan Bi, Qifeng Wang, Zhouguang Hui

**Affiliations:** 1 Department of Radiation Oncology, National Clinical Research Center for Cancer/Cancer Hospital, National Cancer Center, Chinese Academy of Medical Sciences and Peking Union Medical College, Panjiayuan Nanli #17, Chaoyang District, Beijing, China; 2 Department of Radiation Oncology, Radiation Oncology Key Laboratory of Sichuan Province, Sichuan Clinical Research Center for Cancer, Sichuan Cancer Hospital & Institute, Sichuan Cancer Center, Affiliated Cancer Hospital of University of Electronic Science and Technology of China, No.55.Section 4, South Renmin Road, Chengdu, 610042 China; 3 Department of Pathology, National Clinical Research Center for Cancer/Cancer Hospital, National Cancer Center, Chinese Academy of Medical Sciences and Peking Union Medical College, Panjiayuan Nanli #17, Chaoyang District, Beijing, China; 4 Department of Diagnostic Radiology, Radiation Oncology Key Laboratory of Sichuan Province, Sichuan Clinical Research Center for Cancer, Sichuan Cancer Hospital & Institute, Sichuan Cancer Center, Affiliated Cancer Hospital of University of Electronic Science and Technology of China, No.55.Section 4, South Renmin Road, Chengdu, China; 5 Department of VIP Medical Services & Radiation Oncology, National Clinical Research Center for Cancer/Cancer Hospital, National Cancer Center, Chinese Academy of Medical Sciences and Peking Union Medical College, Panjiayuan Nanli #17, Chaoyang District, Beijing, 100021 China; 6 Department of Diagnostic Radiology, National Clinical Research Center for Cancer/Cancer Hospital, National Cancer Center, Chinese Academy of Medical Sciences and Peking Union Medical College, Panjiayuan Nanli #17, Chaoyang District, Beijing, China; 7 Department of Thoracic Surgery, National Clinical Research Center for Cancer/Cancer Hospital, National Cancer Center, Chinese Academy of Medical Sciences and Peking Union Medical College, Panjiayuan Nanli #17, Chaoyang District, Beijing, China

**Keywords:** Esophageal neoplasms, Magnetic resonance, Treatment outcome, Neoadjuvant Chemoradiotherapy

## Abstract

**Background:**

More than 40% of patients with resectable esophageal squamous cell cancer (ESCC) achieve pathological complete response (pCR) after neoadjuvant chemoradiotherapy (nCRT), who have favorable prognosis and may benefit from an organ-preservation strategy. Our study aims to develop and validate a machine learning model based on MR radiomics to accurately predict the pCR of ESCC patients after nCRT.

**Methods:**

In this retrospective multicenter study, eligible patients with ESCC who underwent baseline MR (T2-weighted imaging) and nCRT plus surgery were enrolled between September 2014 and September 2022 at institution 1 (training set) and between December 2017 and August 2021 at institution 2 (testing set). Models were constructed using machine learning algorithms based on clinical factors and MR radiomics to predict pCR after nCRT. The area under the curve (AUC) and cutoff analysis were used to evaluate model performance.

**Results:**

A total of 155 patients were enrolled in this study, 82 in the training set and 73 in the testing set. The radiomics model was constructed based on two radiomics features, achieving AUCs of 0.968 (95%CI 0.933–0.992) in the training set and 0.885 (95%CI 0.800-0.958) in the testing set. The cutoff analysis resulted in an accuracy of 82.2% (95%CI 72.6-90.4%), a sensitivity of 75.0% (95%CI 58.3-91.7%), and a specificity of 85.7% (95%CI 75.5-96.0%) in the testing set.

**Conclusion:**

A machine learning model based on MR radiomics was developed and validated to accurately predict pCR after nCRT in patients with ESCC.

**Supplementary Information:**

The online version contains supplementary material available at 10.1186/s40644-024-00659-x.

## Background

Neoadjuvant chemoradiotherapy (nCRT) combined with surgery has successfully improved the survival of patients with resectable esophageal cancer and has become a standard treatment [1]. Two large randomized control trials, CROSS and NEOCRTEC5010, showed that 43.2–49% of patients with esophageal squamous cell carcinoma (ESCC) were confirmed as pathological complete response (pCR) in surgical resection specimens after nCRT [2, 3], which is related to favorable prognosis [4]. Esophagectomy is known to have a high incidence of surgical complications and a significant decrease in the quality of life [5, 6]. Active surveillance is an organ-preserving strategy in which patients predicted to reach pCR after nCRT continue surveillance rather than surgery. Researches indicate it achieves similar disease control and survival compared to esophagectomy after nCRT [7]. The success of this strategy hinges on accurately predicting pCR after nCRT to select suitable patients who can benefit from organ preservation and to ensure the efficacy of treatment in patients with residual disease. Therefore, prediction of pCR is important for the individualized treatment of ESCC.

Previous studies have explored the prediction of nCRT response for esophageal cancer by conventional evaluation methods based on CT, PET/CT, or endoscopic biopsy. However, the accuracy of these methods is limited because of the difficulty in distinguishing tumor tissues from reactive changes [8]. Radiomics, which extracts numerous features from images, has shown promising results in predicting pCR in esophageal cancer after nCRT based on CT or PET/CT, with reported areas under the curve (AUCs) of 0.65–0.85 in testing sets [9–13].

MR has high tissue resolution, contains a tremendous amount of information, and has been improved to have superior sensitivity relative to CT and PET in diagnostic performance for esophageal cancer [14]. Conventional MR image analysis has shown promising potential in predicting pCR after nCRT in esophageal cancer. A meta-analysis including seven studies with 158 patients showed that the increase of apparent diffusion coefficient (ADC) computed from diffusion-weighted imaging (DWI) during nCRT was significantly different between pCR and non-pCR groups [15]. However, no study has explored MR radiomics, which can maximize the use of valuable image information, to predict nCRT response in esophageal cancer. In this context, we aimed to develop an accurate early prediction model using a machine learning method based on MR radiomics to predict pCR after nCRT in ESCC.

## Methods

### Patients

The institutional review boards approved this retrospective study of the two institutions, and the requirement for informed consent was waived.

Consecutive patients with ESCC were included between September 2014 and September 2022 at Institution 1 (National Cancer Center, Beijing, China) and between December 2017 and August 2021 at Institution 2 (Sichuan Cancer Hospital & Institution, Sichuan, China). The inclusion criteria were as follows: (1) histologically confirmed ESCC; (2) underwent neoadjuvant concurrent chemoradiation followed by radical esophagectomy; (3) age 18–80 years; (4) Karnofsky performance status ≥ 70; and (5) underwent pretreatment fat-suppressed T2-weighted imaging (T2WI). Patients were excluded if they had any of the following conditions: (1) distant metastasis (except for supraclavicular lymph node metastasis) or (2) insufficient T2WI quality (with MR artifacts existing inside the primary tumor).

Pretreatment staging started with an in-depth medical history and thorough physical examination. Patients then underwent a series of diagnostic evaluations, including routine hematologic and biochemical tests, enhanced CT scans of the neck, thorax, and abdomen, upper gastrointestinal endoscopy with biopsy, and endoscopic ultrasonography. Cervical ultrasonography, augmented by fine-needle aspiration, was employed when lymph node involvement was suspected. Additional diagnostic procedures, such as bronchoscopy, positron emission tomography (PET) and radionuclide bone imaging, were optional if clinically indicated. The details of the neoadjuvant chemoradiotherapy regimens are provided in eAppendix 1. Before surgery, clinical restaging was performed through upper gastrointestinal endoscopy with endoscopic ultrasonography and neck-thorax-abdomen CT. PET was indicated if distant progression was suspected. Patients at institutions 1 and 2 were allocated to the training and testing sets, respectively.

### Pathological assessment

Two senior pathologists (L. X. and Z. Y.) specializing in esophageal cancer with more than 10 years of experience performed pathological assessments. Pathological assessment included detailed evaluations of the tumor, such as its location, type, and histological grade, along with the depth of tumor invasion and the status of resection margins. The tumor’s response to treatment was assessed using the Mandard tumor regression grade (TRG) system. Additionally, lymph node status was thoroughly examined, including the location, number of nodes affected, and the extent of therapeutic response in these nodes. The pCR was defined as the absence of residual tumor cells in all resected specimens, including the primary tumor site and lymph nodes.

### MRI protocol

Patients received baseline MR scan in 2 weeks before nCRT. Fat-suppressed T2WI were accessed from two institutions. At institution 1, all MR examinations were performed using a GE Discovery MR750w 3.0T scanner or a GE Discovery MR750 3.0T scanner. At institution 2, MR examinations were performed using four scanners (SIEMENS Skyra 3.0T scanner, SIEMENS Avanto 1.5T scanner, UIH uMR780 3.0T scanner, and UIH uMR588 1.5T scanner). Details of all scanner protocols are provided in eTable 1.

### Segmentation of regions of interest (ROIs)

The 3D-ROIs of the primary tumor were manually segmented on axial T2WI independently at the two institutions. Images of patients in the training set were segmented using ITK-SNAP software by one radiologist (Y. L.) with 4 years of experience and reviewed by a senior radiologist (Z. H.) with more than 20 years of experience who was blinded to the pathological information. Images of patients in the testing set were segmented using MIM software by one radiologist (H. Z.) with four years of experience and reviewed by a senior radiologist (Y. W.) with 14 years of experience in a blinded manner. Disagreements were resolved through discussion until a consensus was reached. The segmentation of 10 randomly selected patients from the training set was performed by another radiologist (Z. M.) with five years of experience. Inter-class correlation coefficients (ICCs) were calculated using the two sets of segmentation in the training set and features with ICCs greater than 0.75 were considered satisfactory reproducibility for further analysis.

### Radiomics feature extraction

The radiomics features from ROIs were computed using the PyRadiomics package (version 3.0.1), as recommended by the IBSI [16]. First, the z-score method was used to normalize the distribution of image densities. The voxel size was resampled to 1 mm ×1 mm ×1 mm. Features were extracted from the original, wavelet-filtered, and Laplacian of Gaussian-filtered images. Features with satisfactory reproducibility were harmonized in the two institutions using the ComBat method to compensate for multivendor effects [17]. Further details on the definitions and algorithms of radiomics features are provided in eAppendix 2.

### Feature selection and modeling

Feature selection was performed independently in the training set. Clinical factors were selected using recursive feature elimination with 5-fold cross-validation (RFECV). The RFECV method used backward elimination to iteratively remove the feature with the least contribution to the predictive performance of the classifier. It generated rankings of features based on the number of iterations when the feature was removed. This process continued until the model performance became worse in the cross-validation, leaving the most important features.

Radiomics features with satisfactory reproducibility were selected using Pearson correlation analysis (|r| threshold of 0.95) to eliminate redundancy. RFECV was then performed to select the optimal features from the remaining features for radiomics model construction.

**Fig. 1:**
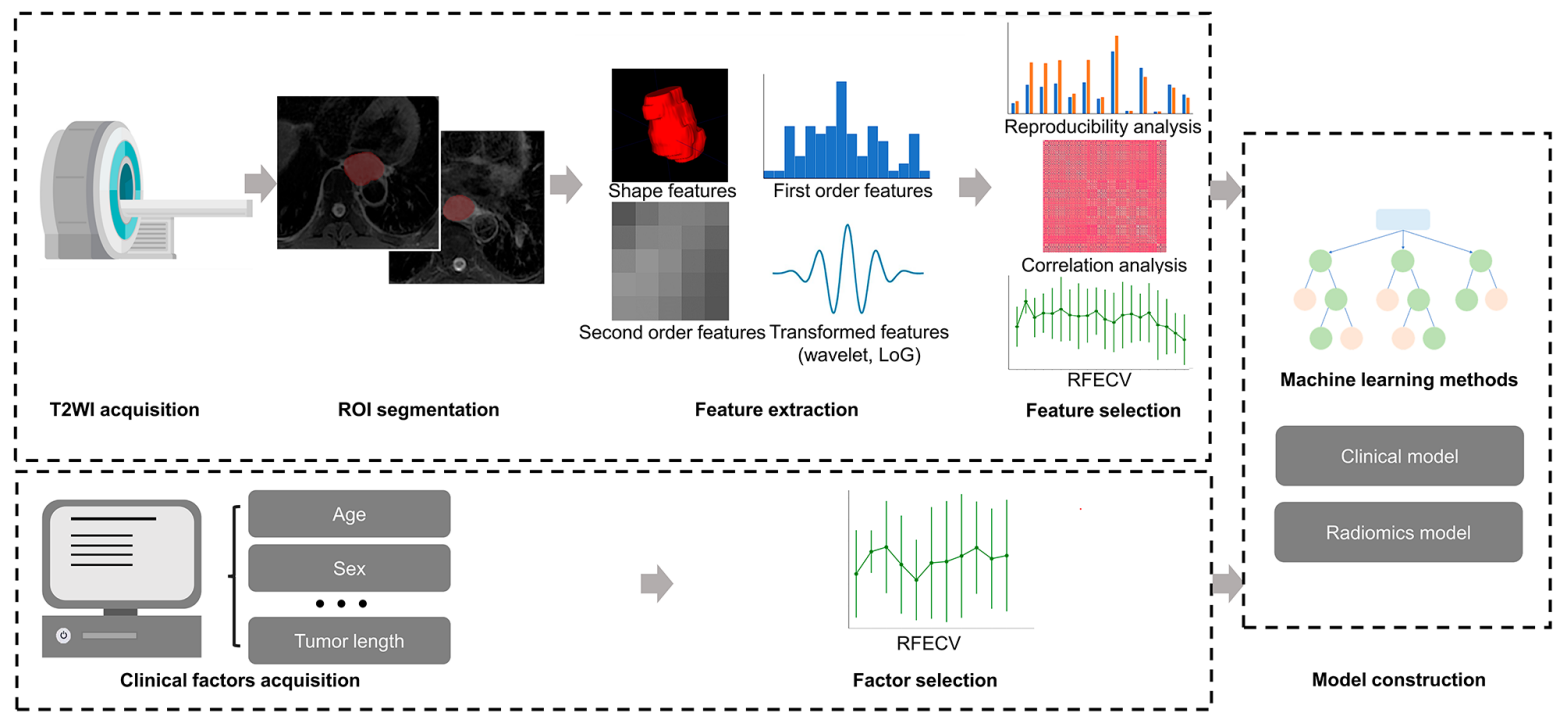
Analysis flowchart. T2WI, T2-weighted imaging; ROI, region of interest; RFECV, recursive feature elimination with cross validation

Logistic Regression, K-Nearest Neighbor, Support Vector Machine, Decision Tree, Random Forest and XGBoost were adopted as the machine learning algorithms for model construction on the training set. The hyperparameters were tuned using the grid-search method in the training set. Internal five-fold cross-validation were performed in the training set to generate mean AUCs. The constructed models were applied independently to the testing set. The workflow of model development is shown in Fig. 1.

### Statistical analysis

The receiver operating characteristic (ROC) curve and AUC were used to evaluate the model performance. The cutoffs were determined using the Youden index in the training set. They applied to the testing set to calculate the accuracy, sensitivity, specificity, positive predictive value (PPV), and negative predictive value (NPV). We utilized 1000 bootstraps to generate a 95% confidence interval (95%CI). A decision curve analysis was performed to evaluate the benefits of the models in clinical applications. The DeLong and integrated discrimination improvement (IDI) tests were performed to compare the predictive performances of different models.

Statistical analyses were performed using SPSS version 26.0, Python version 3.9, and R version 4.1.2. Categorical variables were compared using Fisher’s exact test, and continuous variables were compared using the Mann–Whitney U test. Kaplan-Meier analysis was utilized to evaluate the disease-free survival (DFS) and log-rank tests were performed between pCR group and non-pCR group, in actual population and predicted population, respectively. Statistical tests were two-tailed, and *P* < 0.05 was considered significant. The data processing and model construction code is publicly available (https://github.com/NCCYUNSONG/ESO_MR_RESEARCH.git).

## Results

### Patient characteristics

**Fig. 2:**
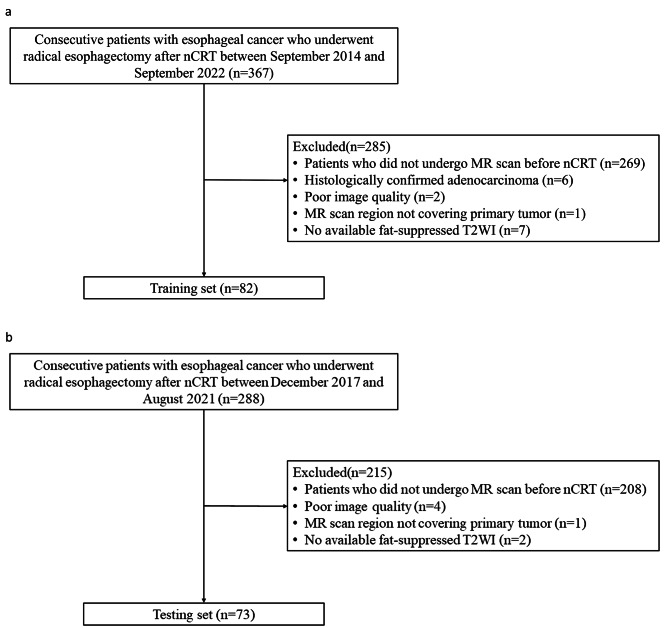
**a**, Selection process of institution 1 (training set); **b**, Selection process of institution 2 (testing set). nCRT, neoadjuvant chemoradiotherapy; T2WI, T2-weighted imaging

A total of 655 patients were screened, 155 of whom were included in this study. A flowchart of patient selection is shown in Fig. 2. Eighty-two patients (66 men and 16 women, median age 62 years [IQR 55–66]) and 73 patients (61 men and 12 women, median age 62 years [IQR 55–67]) were eligible for the training (institution 1) and testing sets (institution 2), respectively. The median radiation doses were 41.4 Gy (IQR 37.8–41.4) in the training set and 40.0 Gy (IQR 40.0–40.0) in the testing set. Sixty-three (76.8%) patients in the training set received simultaneous integrated boost of PGTV (median dose 47.5 Gy, IQR [44.9–49.2]), which was not applied to patients in the testing set. The intervals between nCRT and surgery were 57 days (IQR 48–79) and 47 days (IQR 40–52) in the training and testing sets, respectively. Thirty-nine (47.5%) patients achieved pCR in the training set, and 24 (32.9%) patients achieved pCR in the testing set. The patients’ clinical characteristics are presented in Table 1.

**Table 1:** Patient Characteristics

Characters	Institution 1 (training set) *N* = 82	Institution 2 (testing set) *N* = 73	*P*-value
pCR	39 (47.5)	24 (32.9)	0.073
Age, years (median, IQR)	62 (56, 66)	62 (55, 67)	0.836
Sex			0.304
Female	16 (19.6)	11 (15.1)	
Male	66 (80.4)	62 (84.9)	
KPS			< 0.001
≤ 80	48 (58.5)	13 (17.8)	
> 80	34 (41.5)	60 (82.2)	
Location			0.935
Neck	0 (0.0)	1 (1.4)	
Upper thoracic	9 (11.0)	7 (9.6)	
Middle thoracic	29 (35.4)	28 (38.3)	
Lower thoracic	42 (51.2)	36 (49.3)	
Gastroesophageal junction	2 (2.4)	1 (1.4)	
Length, cm (median, IQR)	5 (4.0, 6.2)	5 (4.0, 7.0)	0.461
cT			0.781
1	1 (1.2)	1 (1.4)	
2	4 (4.9)	3 (4.1)	
3	53 (64.6)	53 (72.6)	
4	24 (29.3)	16(21.9)	
cN			0.173
0	9 (11.0)	2 (2.7)	
1	22 (26.8)	27 (37.0)	
2	42 (51.2)	36 (49.3)	
3	9 (11.0)	8 (11.0)	
cM			0.030
0	76 (92.7)	73 (100)	
1	6 (7.3)	0 (0.0)	
Chemotherapy regimen			0.009
Platinum based	62 (75.6)	66 (90.4)	
Non-platinum based	20 (24.4)	7 (9.6)	
Radiotherapy technique			< 0.001
IMRT	13 (15.9)	73 (100)	
VMAT	69 (84.1)	0 (0.0)	
Radiation dose, Gy (median, IQR)	41.4 (37.8, 41.4)	40.0 (40.0, 40.0)	0.142
Simultaneous integrated boost	63 (76.8)	0 (0.0)	< 0.001
Interval between nCRT and surgery, days (median, IQR)	57 (48, 79)	47 (40, 52)	< 0.001

Data are presented as n (%) unless otherwise stated. pCR, pathological complete response; IQR, interquartile range; KPS, Karnofsky Performance Status; cT, clinical T stage; cN, clinical N stage; cM, clinical M stage; IMRT, Intensity-Modulated Radiation Therapy; VMAT, Volumetric Modulated Arc Therapy; nCRT, neoadjuvant chemoradiotherapy

There were no statistically significant differences in the clinical characteristics between pCR and non-pCR patients in the training and testing sets, except for sex (*P* = 0.03) and tumor location (*P* = 0.045) in the testing set (eTable 2).

### Performance of the clinical model

**Fig. 3:**
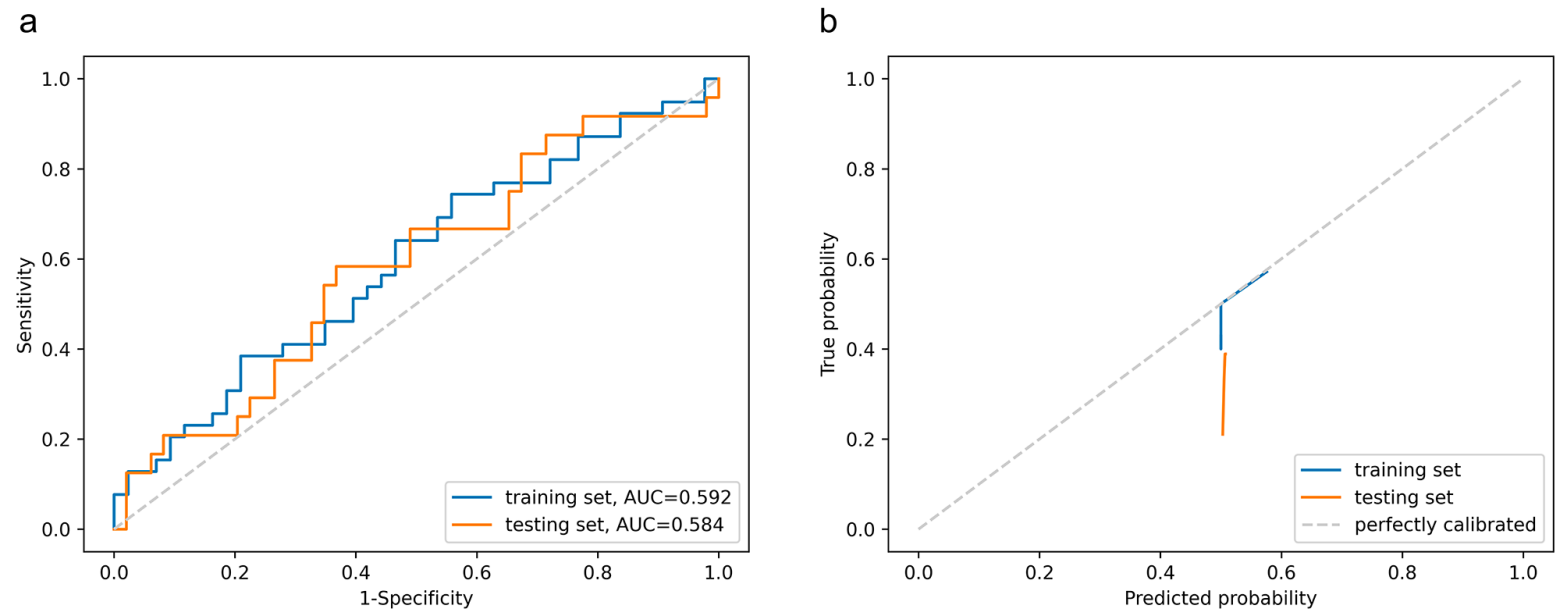
Performance of the clinical model. **a**, Receiver operating characteristic curves; **b**, Calibration curves. AUC, area under the curve

The candidates for clinical factors were the same as those listed in Table 1. Age, tumor length, and interval between nCRT and surgery were included in the model after filtration (eFigure 1a). The best model using logistic regression (eTable 3) achieved AUCs in the training and testing set being 0.592 (95%CI 0.472–0.716) and 0.584 (95%CI 0.441–0.714), respectively (Fig. 3a; Table 2). The accuracies were 58.5% (95%CI 53.7-70.7%) and 61.6% (95%CI 43.8-75.3%) in the training and testing sets, respectively. Internal cross-validation showed a mean AUC of 0.596 (eFigure 2a).

**Table 2:** Predictive performance of final models

	AUC	Accuracy (%)	Sensitivity (%)	Specificity (%)	PPV (%)	NPV (%)
**Clinical model**						
Training set	0.592 (0.472–0.716)	58.5 (48/82) [53.7–70.7]	74.4 (29/39) [12.8–94.9]	44.1 (19/43) [20.9–100]	54.7 (29/53) [51.0-100]	65.5 (19/29) [55.3–85.7]
Testing set	0.584 (0.441–0.714)	61.6 (45/73) [43.8–75.3]	58.3 (14/24) [16.7–100]	63.3 (31/49) [20.4–98.0]	43.8 (14/32) [36.1–87.5]	75.6 (31/41) [70.3–100]
**Radiomics model**						
Training set	0.968 (0.933–0.992)	92.7 (76/82) [87.8–97.6]	87.2 (34/39) [76.9–97.4]	97.7 (42/43) [93.0-100.0]	97.1 (34/35) [91.4–100.0]	89.4 (42/47) [82.4–97.6]
Testing set	0.885 (0.800-0.958)	82.2 (60/73) [72.6–90.4]	75.0 (18/24) [58.3–91.7]	85.7 (42/49) [75.5–96.0]	72.0 (18/25) [58.6–89.5]	87.5 (42/48) [80.0-95.5]

Data in parentheses are numerators and denominators, with 95% CIs in brackets. AUC, area under the curve; PPV, positive predictive value; NPV, negative predictive value

### MR radiomic analysis

In total, 1106 features were extracted from each patient. The details of the extracted features are listed in eTable 4. Satisfactory reproducibility was achieved in 928 (83.9%) of the 1106 features with an ICC threshold of 0.75. After correlation analysis with a Pearson|r| > 0.95 to remove multicollinear features, 299 features remained. The top two features, which were both wavelet features, of the ranking by RFECV were finally selected to build the radiomics model (eFigure 1b, eTable 4).

**Fig. 4:**
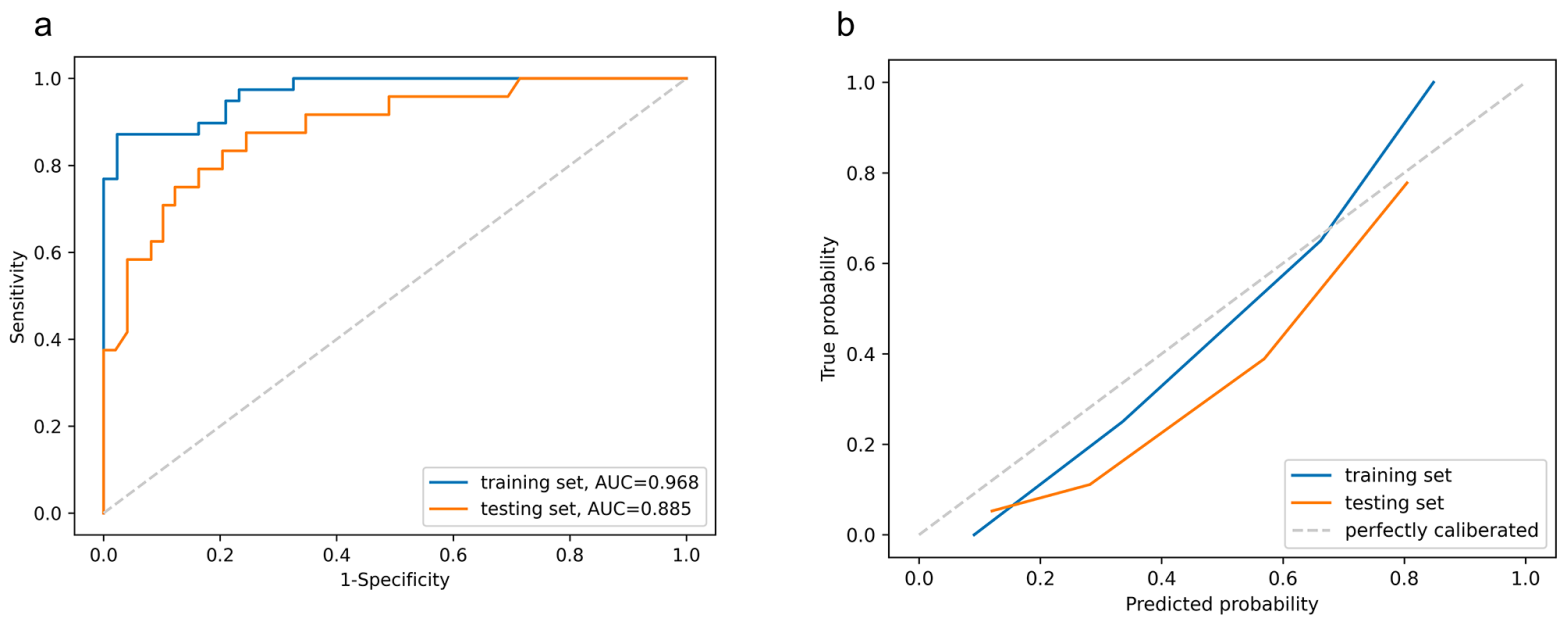
Performance of the radiomics model. **a**, Receiver operating characteristic curves; **b**, Calibration curves. AUC, area under the curve

The best radiomics model was constructed using Random Forest (eTable 3), with AUCs of 0.968 (95%CI 0.933–0.992) in the training set and 0.885 (95%CI 0.800-0.958) in the testing set, respectively (Fig. 4a; Table 2). Accuracies were 92.7% (95%CI 87.8-97.6%) and 82.2% (95%CI 72.6-90.4%), respectively. In the training set, the sensitivity, specificity, PPV, and NPV were 87.2% (95%CI 76.9-97.4%), 97.7% (95%CI 93.0-100%), 97.1% (95%CI 91.4-100%), and 89.4% (95%CI 82.4-97.6%), respectively. In the testing set, the sensitivity, specificity, PPV and NPV were 75.0% (95%CI 58.3-91.7%), 85.7% (95%CI 75.5-96.0%), 72.0% (95%CI 58.6-89.5%), and 87.5% (95%CI 80.0-95.5%), respectively (Table 2). Internal cross-validation showed a mean AUC of 0.805 (eFigure 2b). Satisfactory calibration was achieved (Fig. 4b), and decision curve analysis confirmed the clinical benefit of the radiomics model (eFigure 3). The predictive performance of the radiomics model in both training and testing set was significantly higher than those of the clinical model using the DeLong (*P* < 0.001 in training and testing sets) and IDI (*P* < 0.001 in the training set and *P* = 0.001 in the testing set) tests (Table 3).

**Table 3:** Comparison of predictive performance of models

Model comparison	DeLong test *P*-value	IDI test *P*-value
Radiomics model (Training set) vs. Clinical model (Training set)	< 0.001	< 0.001
Radiomics model (Testing set) vs. Clinical model (Testing set)	< 0.001	0.001

IDI, integrated discrimination improvement

The analysis of radiomics features across various scanning parameters revealed that the selected features demonstrated commendable stability, exhibiting no statistically significant differences when subjected to multiple parameters (eTable 6). Furthermore, the radiomics model exhibited robust performance across distinct scanning parameters, achieving AUCs of 1.000 and 0.971 in the training set, and 0.891, 1.000, and 0.833 in the testing set, for different scanning parameters (eFigure 5).

Correlation analysis revealed that the two chosen radiomics features had a significant association with clinical T stage (*P* = 0.031) and a marginally significant relationship with clinical N stage (*P* = 0.067), respectively (eFigure 5).

With a median follow-up of 32 months (IQR 16–46) in the training set and 24 months (IQR 16–32) in the testing set, the radiomics model’s predicted pCR group showed significantly longer DFS than the predicted non-pCR group in both training and testing sets, consistent with the actual pCR and non-pCR groups (all *P* < 0.05) (eFigure 6).

## Discussion

A machine learning model based on MR radiomics was developed to predict pCR in patients with ESCC after nCRT precisely. The model demonstrated satisfactory predictive performance in the external testing set with high AUC, sensitivity, and specificity, which can assist in the implementation of individualized treatment for ESCC. To the best of our knowledge, this is the first study to use MR radiomics and the first to evaluate the predictive value of MR in an external testing set, in predicting pCR in esophageal cancer after nCRT.

In this study, we established a radiomics model using pretreatment T2WI of patients with ESCC after nCRT to predict pCR, with an AUC of 0.885 (95%CI 0.800-0.958) in the external testing set. The performance of the radiomics model was significantly better than that of the clinical model. Previous studies have also shown that clinical factors cannot accurately predict pCR after nCRT for esophageal cancer [18, 19] and are generally not included in the final models of radiomics studies [31]. Similarly, the clinical model established using demographic, tumor, and treatment-related features showed poor predictive ability (AUC 0.584, 95%CI 0.441–0.714).

The radiomics model developed in this study outperforms those reported in similar studies. Previous studies focusing on the value of radiomics have primarily used CT and PET/CT to predict pCR after nCRT for esophageal cancer. For example, van Rossum et al. [13] used PET images from 217 patients with esophageal cancer to extract radiomic features before and after nCRT, achieving an AUC of 0.77 in internal testing. Yang et al. [12] used pretreatment CT images to build a radiomics model to predict pCR, achieving an AUC of 0.79 in the testing set. However, these studies lacked external testing, limiting their results’ generalizability. Hu et al. [9] conducted a multicenter study, building a radiomics model with CT images of 161 patients in the training set to predict pCR in an external testing set of 70 patients with an AUC of 0.852. However, imaging protocols were limited, which could affect their generalizability. MR has high soft tissue resolution and abundant signal information. A meta-analysis showed that the pooled sensitivity and specificity of MR in predicting pCR for esophageal cancer after nCRT were 80% and 83%, respectively. In contrast, those of CT were 35% and 83%, and those of PET were 62% and 73%, respectively [8]. Li et al. [20] built radiomics models for predicting the treatment response in patients with colorectal cancer after neoadjuvant chemotherapy, achieving an AUC of 0.766 for CT and 0.859 for T2WI in the testing set. Fat-suppressed T2WI has the advantages of delineating the tumor from surrounding fat and providing better information about the tumor’s extension, which is indicated to lead to better evaluation of tumors compared to conventional T2WI [21]. Hou et al. [22] compared radiomics based on fat-suppressed T2WI and conventional T2WI in predicting response to nCRT according to RECIST in ESCC and found fat-suppressed T2WI leading to higher accuracy. In our study, the MR radiomics-based model based on fat-suppressed T2WI achieved accurate prediction (AUC = 0.885) in the external testing set of 73 patients with images obtained from four different MR scanners, and satisfactory performances were achieved separately for different scanners, demonstrating excellent predictive performance and generalizability compared to previous studies, which is potentially valuable for clinical application. Additionally, the predicted pCR group exhibited superior DFS relative to the non-pCR group, highlighting the prognostic value of our model. This reinforces its potential as an effective aid in clinical decision-making processes.

Our model expands the domain of predictive models and demonstrates better accuracy, although a few studies have shown promising potential for MR-based prediction of neoadjuvant therapy efficacy in esophageal cancer. In a prospective study, the ADC was used to predict pCR after nCRT for esophageal cancer, achieving an AUC of 0.791 [23]. Meta-analysis revealed that changes of ADC during treatment significantly associated with pCR after neoadjuvant therapy for esophageal cancer [15]. However, baseline ADC was not associated with pathological response, indicating the ADC may not be competent for early prediction [15]. Besides, the lack of testing sets in these studies limits their reliability. Vollenbrock et al. [24] investigated the accuracy of qualitative assessment using T2WI and DWI, with an AUC of 0.7. Nevertheless, although it is valuable to predict pCR based on DWI, small sample size, lacking of external validation and various cut-offs of ADC in those studies limit its clinical applicability and the precision is highly dependent on multiple time-point imaging. In contrast, our model used pretreatment images to perform an early prediction with high accuracy in both training and testing sets of relatively larger sample sizes. The selected features are associated with clinical T stage and N stage, suggesting that these features may reflect the state of disease progression to some extent. Qu et al. [25] conducted a single-center study using dynamic contrast-enhanced MRI to extract features and predict responders (Mandard TRG 1–2) after neoadjuvant chemotherapy for esophageal cancer, achieving an AUC of 0.86 (95% CI 0.74–0.97) in the testing set. However, neoadjuvant chemotherapy is only recommended for locally advanced esophageal adenocarcinoma [26]. Taken together, these studies and our current research support the value of MR in predicting the efficacy of neoadjuvant therapy for esophageal cancer.

The present study has several limitations. First, the sample size of the training set was relatively small. Future studies with larger sample sizes may improve the model performance. Second, all the patients included in our study had ESCC, and the predictive performance for the adenocarcinoma was unknown. Third, this was a retrospective study conducted at two centers, and the generalizability of the results requires further validation. Fourth, due to the retrospective nature of this study, MR sequences of patients were imbalanced and some valuable sequences such as DWI were not performed in majority of patients. However, building on the findings of our current study, we are going to undertake a prospective study that employs predefined sequences including DWI with various b-values, as well as dynamic contrast-enhanced MR, and the scanning will be conducted at multiple timepoints, to enhance the precision and performance of our model further. Finally, with the increasing research on neoadjuvant immunotherapy for esophageal cancer, it is necessary to conduct large-scale prospective multicenter studies incorporating multiple MR sequences, molecular biomarkers, and gene expression data to establish accurate and generalizable predictive models.

## Conclusions

In conclusion, our study established and validated a machine learning model based on MR radiomics to predict pCR after nCRT for ESCC, providing a helpful tool for response prediction and may potentially assist personalized treatment. Further validation using larger independent datasets is necessary.

### Electronic supplementary material

Below is the link to the electronic supplementary material.

Supplementary Material 1

Supplementary Material 2

## Data Availability

The data used and analyzed during the current study are available from the corresponding authors on reasonable request.

## Abbreviations

nCRT: Neoadjuvant chemoradiotherapy
ESCC: Esophageal squamous cell carcinoma
pCR: Pathological complete response
AUC: Area under the curve
ADC: Apparent diffusion coefficient
DWI: Diffusion-weighted imaging
T2WI: T2-weighted imaging
ROI: Region of interest
RFECV: Recursive feature elimination with 5-fold cross validation
ROC: Receiver operating characteristics
PPV: Positive predictive value
NPV: Negative predictive value
IDI: Integrated discrimination improvement
IQR: Interquartile range
