# Supplementary material for: MR radiomics predicts pathological complete response of esophageal squamous cell carcinoma after neoadjuvant chemoradiotherapy: a multicenter study
Source: Cancer Imaging. 2024 Jan 23;24:16. doi: 10.1186/s40644-024-00659-x (PMC10804642; doi:10.1186/s40644-024-00659-x)
Supplement: Supplementary file 2 — Supplementary Material 2 [file 40644_2024_659_MOESM2_ESM.docx]

**eAppendix 1. Neoadjuvant chemoradiotherapy regimens**

At institution 1, chemotherapy regimens were as follows: paclitaxel plus platinum (cisplatin, carboplatin, nedaplatin or lobaplatin) weekly for 4-5 cycles or every 3 weeks for 2 cycles; oral 5-fluorouracil based regimen combined with or without every-3-week oxaliplatin for 2 cycles or with weekly nimotuzumab for 4-5 cycles. Radiotherapy was administered using Intensity-Modulated Radiation Therapy (IMRT) or Volumetric Modulated Arc Therapy (VMAT) technique, with prescribed dose of 2Gy per fraction (GTV) by 20~25 fractions or 2.14Gy (PGTV)/1.8Gy (PTV) per fraction by 20~23 fractions.

At institution 2, chemotherapy regimens were as follows: paclitaxel plus platinum (cisplatin, carboplatin, nedaplatin) every 3 weeks for 2 cycles; oral 5-fluorouracil based regimen; oxaliplatin plus capecitabine every 3 weeks for 2 cycles. Radiotherapy was administered using IMRT with prescribed dose of 2Gy per fraction (GTV) by 20~25 fractions.

**eAppendix 2. Details of radiomics features**

Here’s the decription of radiomics features used in our optimal model:

A Gray Level Size Zone (GLSZM) quantifies gray level zones in an image. A gray level zone is defined as a the number of connected voxels that share the same gray level intensity. A voxel is considered connected if the distance is 1 according to the infinity norm (26-connected region in a 3D, 8-connected region in 2D). In a gray level size zone matrix 𝑃(𝑖, 𝑗) the (𝑖, 𝑗) th element equals the number of zones with gray level 𝑖 and size 𝑗 appear in image. Contrary to GLCM and GLRLM, the GLSZM is rotation independent, with only one matrix calculated for all directions in the ROI.

As a two dimensional example, consider the following 5x5 image, with 5 discrete gray levels:


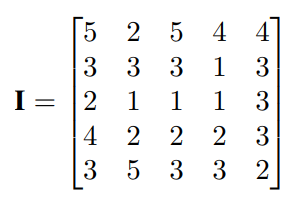


The GLSZM then becomes:


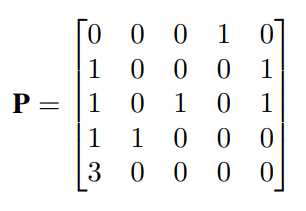


Let:

• 𝑁𝑔 be the number of discrete intensity values in the image

• 𝑁𝑠 be the number of discrete zone sizes in the image

• 𝑁𝑝 be the number of voxels in the image

• 𝑁𝑧 be the number of zones in the ROI, which is equal to $\sum_{i=1}^{Ng} \sum_{j=1}^{Ns} P(i,j)$ and 1 ≤ 𝑁𝑧 ≤ 𝑁𝑝

• P(𝑖, 𝑗) be the size zone matrix

• 𝑝(𝑖, 𝑗) be the normalized size zone matrix, defined as $p\left( i,j \right)= \frac{P(i,j)}{Nz}$

1. Size-Zone Non-Uniformity Normalized (SZNN)


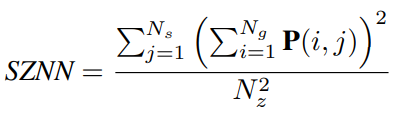


SZNN measures the variability of size zone volumes throughout the image, with a lower value indicating more homogeneity among zone size volumes in the image. This is the normalized version of the SZN formula.

2. Small Area Emphasis (SAE)


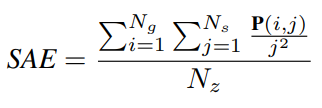


SAE is a measure of the distribution of small size zones, with a greater value indicative of more smaller size zones and more fine textures.

Applying wavelet filter to the input image yields 8 decompositions per level (all possible combinations of applying either a High or a Low pass filter in each of the three dimensions). Here’s the adopted wavelet filters in our optimal model:

1. Wavelet-HLH: This refers to wavelet coefficients obtained by applying High-pass filtering in the first dimension, Low-pass filtering in the second dimension, and High-pass filtering in the third dimension. These coefficients capture the image features that have high frequency in the first and third dimensions and low frequency in the second dimension.

2. Wavelet-LHH: This refers to wavelet coefficients obtained by applying Low-pass filtering in the first dimension, and High-pass filtering in the second and third dimensions. These coefficients capture the image features that have low frequency in the first dimension and high frequency in the second and third dimensions.


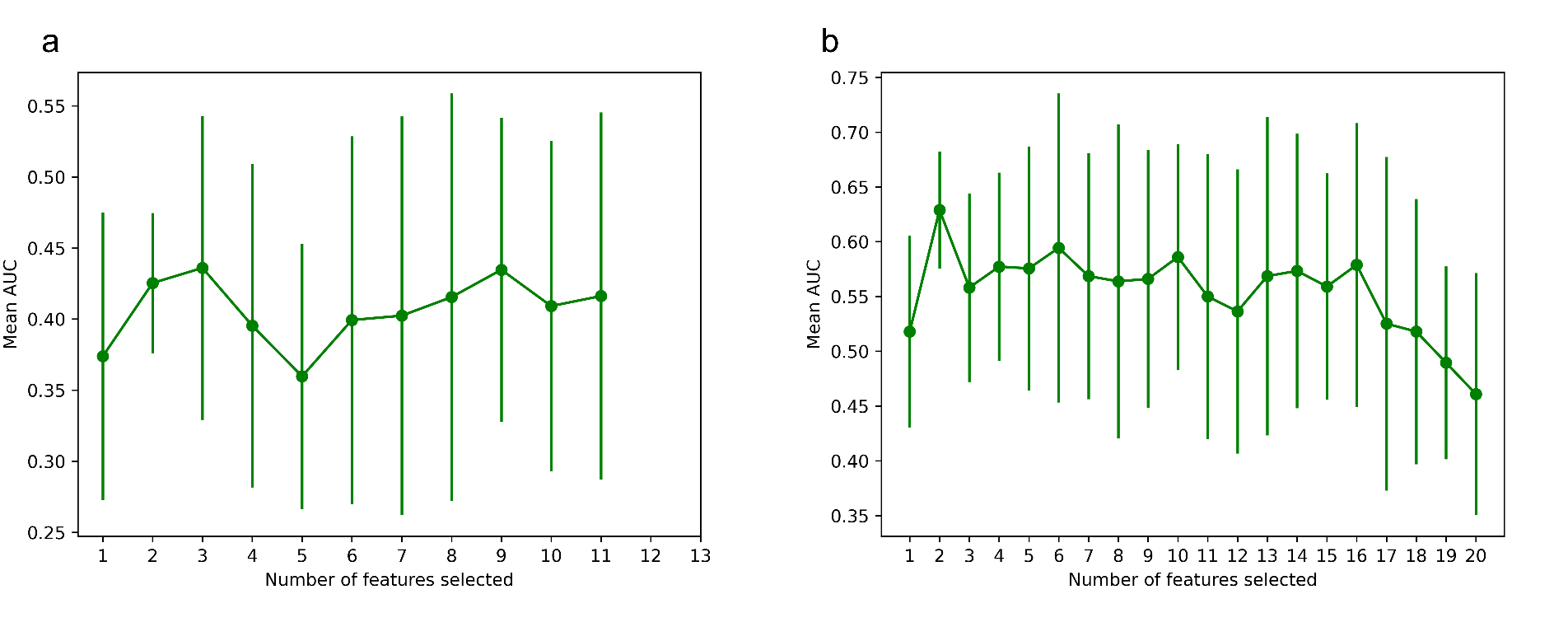


**eFigure 1.** Feature selection process of recursive feature elimination with 5-fold cross validation. Bars on each point represent standard error. a, Selection of clinical factors; b, Selection of radiomics features.


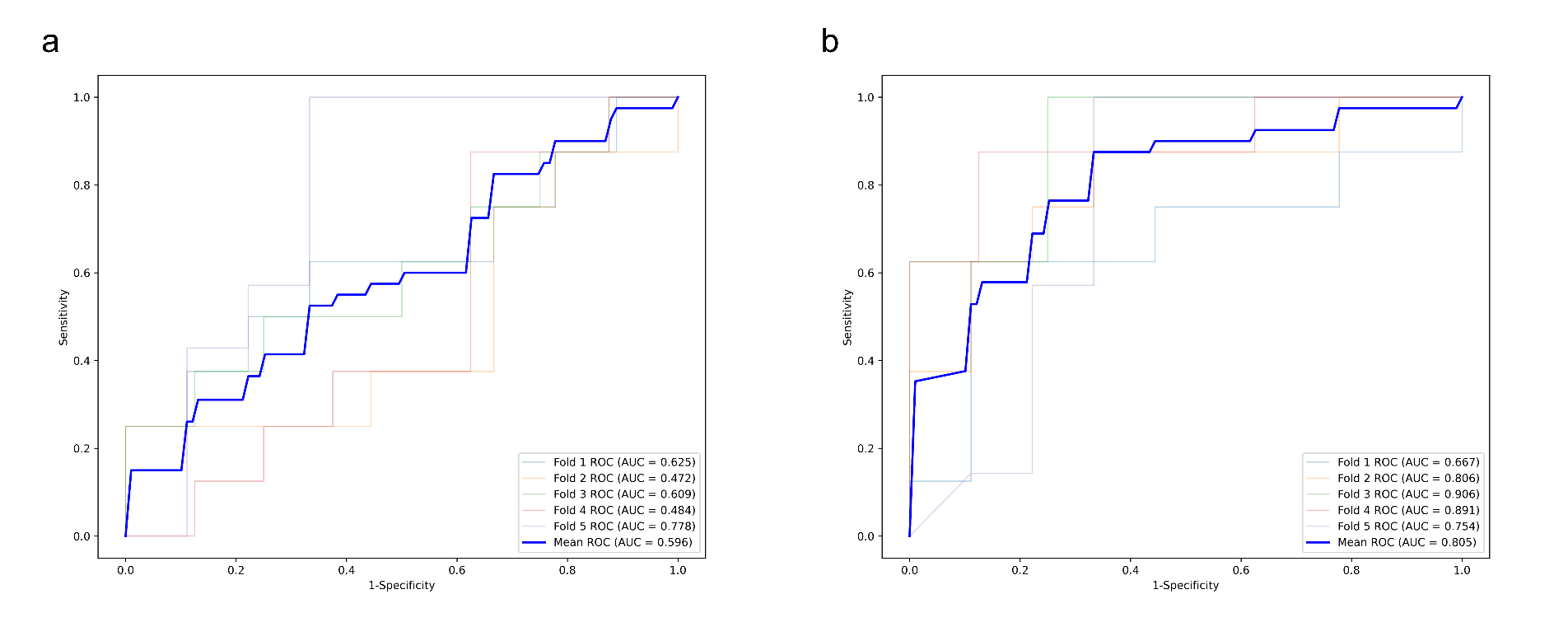


**eFigure 2.** Internal 5-fold cross validation. a, Clinical model; b, Radiomics model. ROC, Receiver operating characteristic curve. AUC, area under the curve.


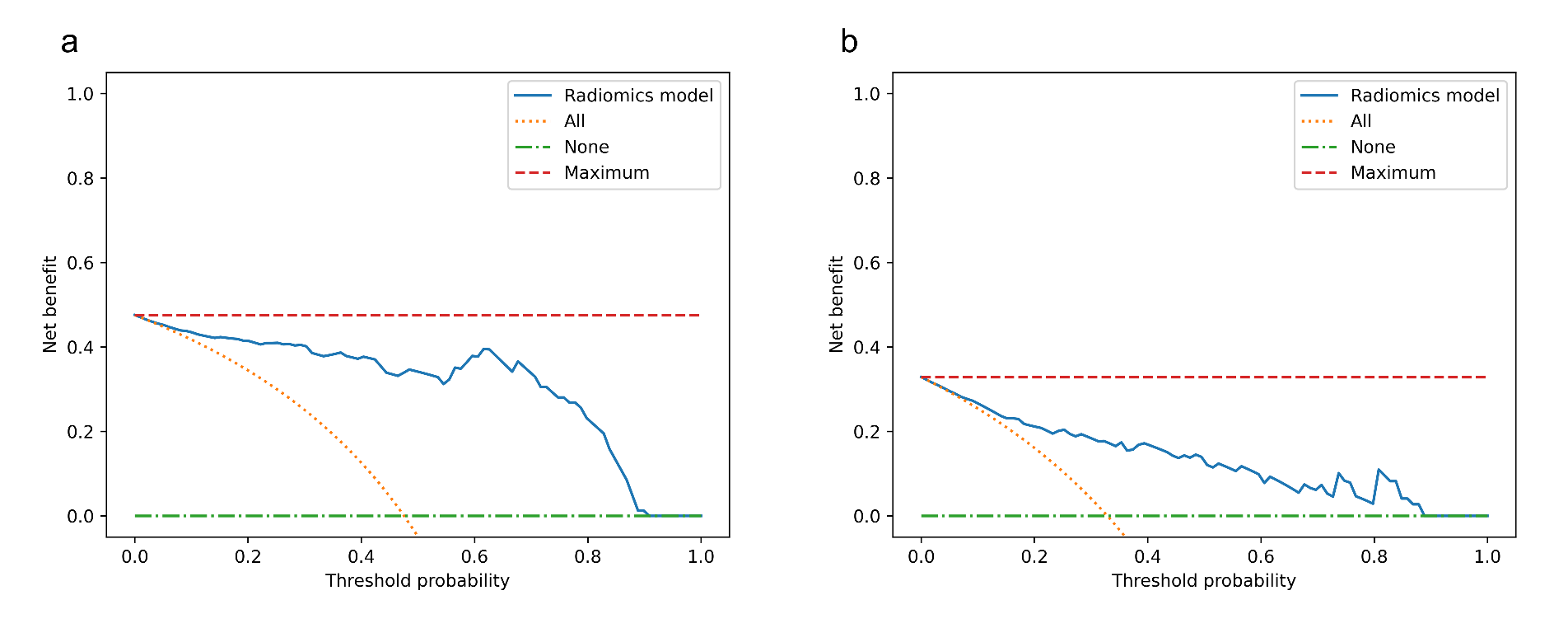


**eFigure 3.** Decision curve analysis. a, Decision curve of the radiomics model in the training set; b, Decision curve of the radiomics model in the testing set.


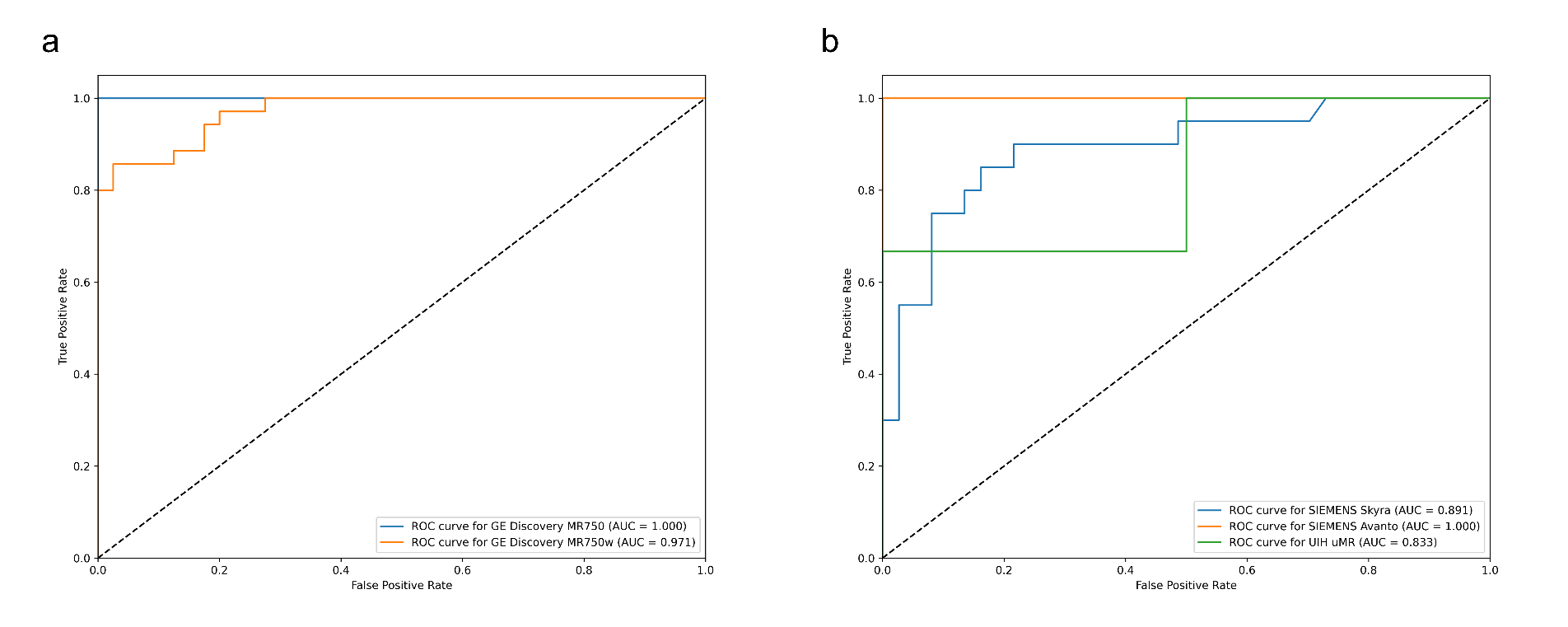


**eFigure 4.** Receiver operating characteristic curves of the radiomics model based on different scanning parameters. a, Performances in the training set; b, Performances in the testing set.


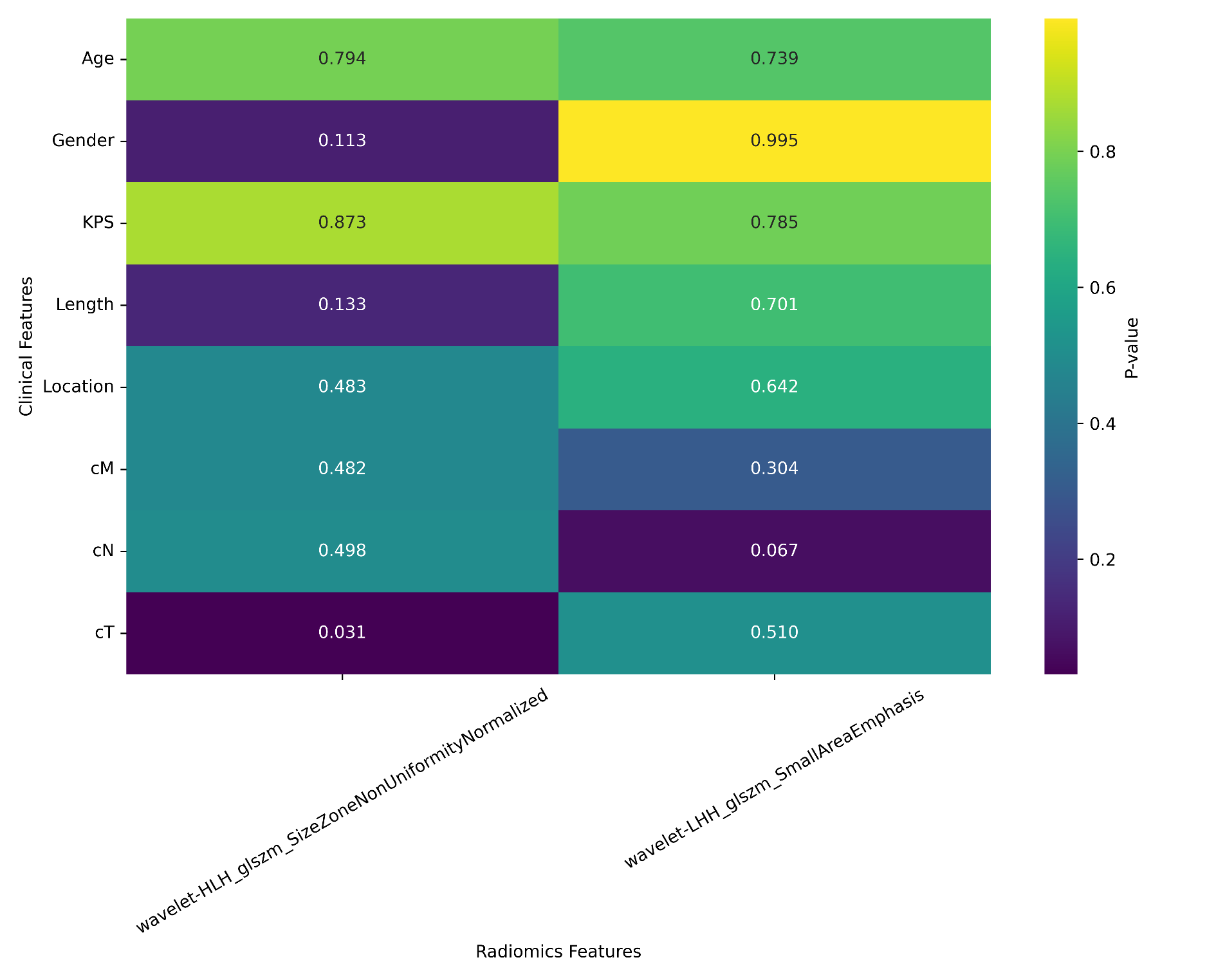


**eFigure 5.** Correlation analysis between radiomics features and clinical factors. KPS, Karnofsky Performance Status; cT, clinical T stage; cN, clinical N stage; cM, clinical M stage.


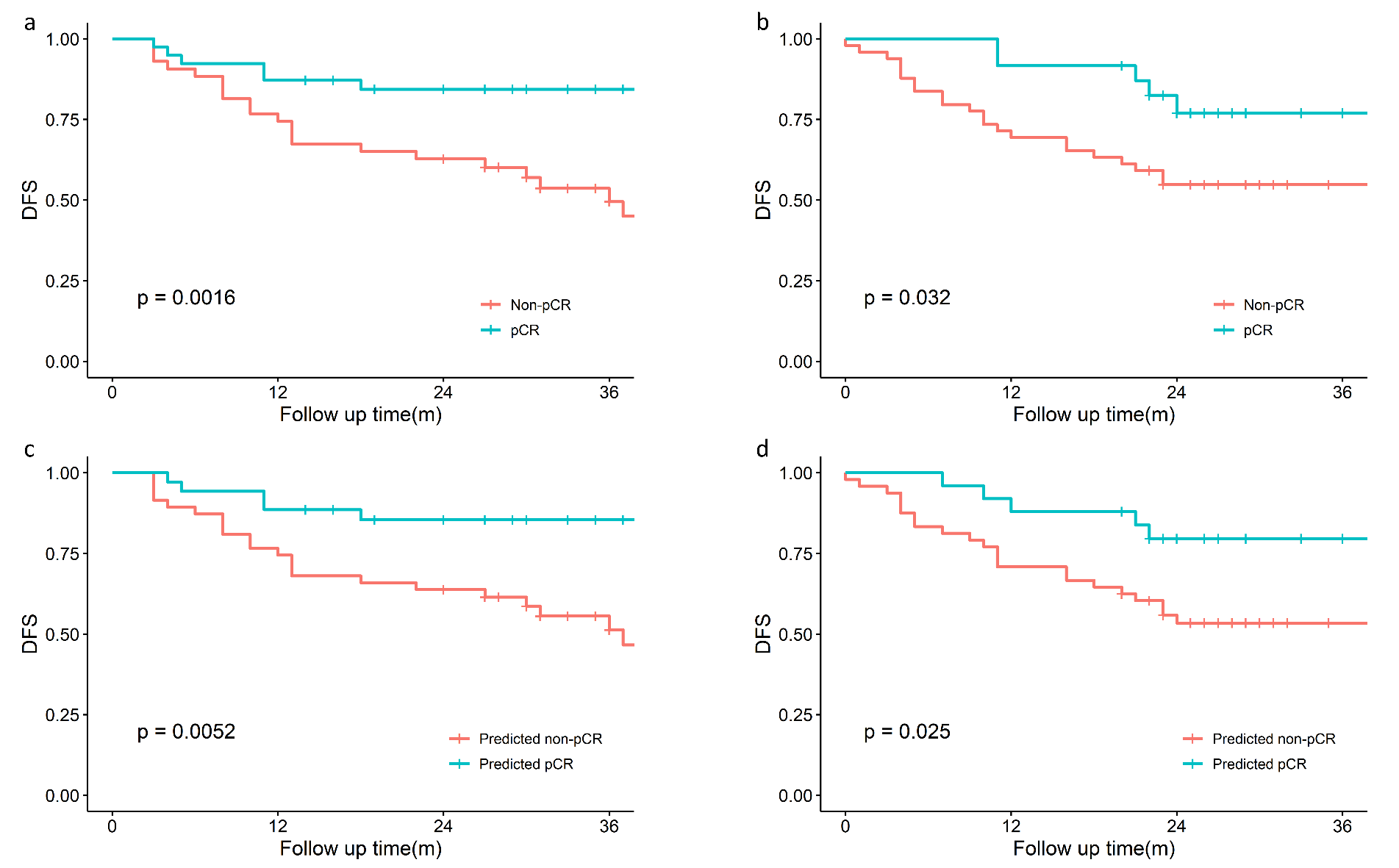


**eFigure 6.** Kaplan-Meier analysis of actual population and predicted population. a, disease-free survival of actual population in training set; b, disease-free survival of actual population in testing set; c, disease- free survival of predicted population in training set; d, disease-free survival of predicted population in testing set. DFS, disease-free survival.

**eTable 1**. T2WI acquisition protocols of MR scanners

| Institution | Scanner | Patients No. | Magnetic field strength | TR (ms) | TE (ms) | Echo train length | Slice thickness | Flip Angle |
| --- | --- | --- | --- | --- | --- | --- | --- | --- |
| Institution 1 | GE Discovery MR750w | 75 | 3.0T | 11250 | 88 | 32 | 5 | 90°/142° |
|  | GE Discovery MR750 | 7 | 3.0T | 18000 | 99 | 32 | 5 | 90° |
| Institution 2 | SIEMENS Skyra | 57 | 3.0T | 4500 | 83 | 43 | 5 | 110° |
|  | SIEMENS Avanto | 9 | 1.5T | 3500 | 85 | 25 | 5 | 140° |
|  | UIH uMR780 | 2 | 3.0T | 4500 | 75 | 28 | 5 | 100° |
|  | UIH uMR588 | 5 | 1.5T | 4000 | 84 | 28 | 5 | 120° |

TR, repetition time; TE, echo time.

**eTable 2**. Association of Patient Characteristics with pCR in training and testing sets.

| Characteristics | Institution 1 (training set) | | P-value | Institution 2 (testing set) | | P-value |
| --- | --- | --- | --- | --- | --- | --- |
|  | pCR (N=39) | Non-pCR (N=43) |  | pCR (N=24) | Non-pCR (N=49) |  |
| Age, years (median, IQR) | 64 (59, 68) | 60 (55, 67) | 0.127 | 62 (56, 67) | 63 (55, 68) | 0.681 |
| Sex |  |  | 0.265 |  |  | 0.033 |
| Female | 10 (25.6) | 6 (14.0) |  | 7 (29.2) | 4 (8.2) |  |
| Male | 29 (74.4) | 37 (86.0) |  | 17 (70.8) | 45 (91.8) |  |
| KPS |  |  | 0.502 |  |  | 0.332 |
| ≤80 | 21 (53.8) | 27 (62.8) |  | 6 (25.0) | 7 (14.3) |  |
| >80 | 18 (46.2) | 16 (37.2) |  | 18 (75.0) | 42 (85.7) |  |
| Location |  |  | 0.696 |  |  | 0.045 |
| Neck | 0 (0.0) | 0 (0.0) |  | 1 (4.2) | 0 (0.0) |  |
| Upper thoracic | 6 (15.4) | 3 (7.0) |  | 3 (12.5) | 4 (8.2) |  |
| Middle thoracic | 14 (35.9) | 15 (34.9) |  | 13 (54.2) | 15 (30.6) |  |
| Lower thoracic | 18 (46.2) | 24 (55.8) |  | 7 (29.2) | 29 (59.2) |  |
| Gastroesophageal junction | 1 (2.6) | 1 (2.3) |  | 0 (0.0) | 1 (2.0) |  |
| Length, cm (median, IQR) | 5.0 (4.0, 6.0) | 5 (4.0, 7.0) | 0.550 | 5.5 (4.0, 7.8) | 5.0 (4.0, 7.5) | 0.917 |
| cT |  |  | 0.249 |  |  | 0.678 |
| 1 | 0 (0.0) | 1 (2.3) |  | 0 (0.0) | 1 (2.0) |  |
| 2 | 2 (5.1) | 2 (4.7) |  | 2 (8.3) | 1 (2.0) |  |
| 3 | 22 (56.4) | 31 (72.1) |  | 17 (70.8) | 36 (73.5) |  |
| 4 | 15 (38.5) | 9 (20.9) |  | 5 (20.8) | 11 (22.4) |  |
| cN |  |  | 0.943 |  |  | 0.586 |
| 0 | 4 (10.3) | 5 (11.6) |  | 1 (4.2) | 1 (2.0) |  |
| 1 | 11 (28.2) | 11 (25.6) |  | 11 (45.8) | 16 (32.7) |  |
| 2 | 19 (48.7) | 23 (53.5) |  | 10 (41.7) | 26 (53.1) |  |
| 3 | 5 (12.8) | 4 (9.3) |  | 2 (8.3) | 6 (12.2) |  |
| cM |  |  | >0.999 |  |  | - |
| 0 | 36 (93.0) | 40 (92.3) |  | 24 (100) | 49 (100) |  |
| 1 | 3 (7.0) | 3 (7.7) |  | 0 (0.0) | 0 (0.0) |  |
| Chemotherapy regimen |  |  | >0.999 |  |  | 0.169 |
| Platinum based | 30 (76.9) | 32 (74.4) |  | 24 (100) | 43 (87.8) |  |
| Non-platinum based | 9 (23.1) | 11 (25.6) |  | 0 (0.0) | 6 (12.2) |  |
| Radiotherapy technique |  |  | >0.999 |  |  | - |
| IMRT | 6 (15.4) | 7 (16.3) |  | 24 (100) | 49 (100) |  |
| VMAT | 33 (84.6) | 36 (83.7) |  | 0 (0.0) | 0 (0.0) |  |
| Radiation dose, Gy (median, IQR) | 41.4 (37.8, 41.4) | 41.4 (37.8, 41.4) | 0.600 | 40 (40, 40) | 40 (40, 40) | 0.101 |
| Simultaneous integrated boost | 28 (71.8) | 35 (81.4) | 0.432 | 0 (0.0) | 0 (0.0) | - |
| Interval between CRT and surgery, days (median, IQR) | 55 (49, 81) | 58 (47,78) | 0.659 | 49 (40, 52) | 47 (39, 54) | 0.859 |

Data are presented as n (%) unless otherwise stated. pCR, pathological complete response; IQR, interquartile range; KPS, Karnofsky Performance Status; cT, clinical T stage; cN, clinical N stage; cM, clinical M stage; IMRT, Intensity-Modulated Radiation Therapy; VMAT, Volumetric Modulated Arc Therapy; nCRT, neoadjuvant chemoradiothera

**eTable 3.** AUCs using different machine learning algorithms

|  | AUC (training set) | AUC (testing set) |
| --- | --- | --- |
| **Clinical model** |  |  |
| Logistic regression | 0.592 | 0.584 |
| Support vector machine | 1.000 | 0.500 |
| K-Nearest Neighbor | 0.615 | 0.500 |
| Decision Tree | 0.997 | 0.496 |
| Random Forest | 1.000 | 0.573 |
| XGBoost | 0.889 | 0.433 |
| **Radiomics model** |  |  |
| Logistic regression | 0.673 | 0.754 |
| Support vector machine | 0.657 | 0.705 |
| K-Nearest Neighbor | 0.856 | 0.730 |
| Decision Tree | 0.968 | 0.770 |
| Random Forest | 0.968 | 0.885 |
| XGBoost | 0.987 | 0.823 |

**eTable 4**. Extracted radiomics features

| Feature groups (n) | Feature names | Feature groups (n) | Feature names |
| --- | --- | --- | --- |
| Shape features (n=14) | shape_Elongation | First-order features (n=18) | firstorder_10Percentile |
|  | shape_Flatness |  | firstorder_90Percentile |
|  | shape_LeastAxisLength |  | firstorder_Energy |
|  | shape_MajorAxisLength |  | firstorder_Entropy |
|  | shape_Maximum2DDiameterColumn |  | firstorder_InterquartileRange |
|  | shape_Maximum2DDiameterRow |  | firstorder_Kurtosis |
|  | shape_Maximum2DDiameterSlice |  | firstorder_Maximum |
|  | shape_Maximum3DDiameter |  | firstorder_MeanAbsoluteDeviation |
|  | shape_MeshVolume |  | firstorder_Mean |
|  | shape_MinorAxisLength |  | firstorder_Median |
|  | shape_Sphericity |  | firstorder_Minimum |
|  | shape_SurfaceArea |  | firstorder_Range |
|  | shape_SurfaceVolumeRatio |  | firstorder_RobustMeanAbsoluteDeviation |
|  | shape_VoxelVolume |  | firstorder_RootMeanSquared |
|  |  |  | firstorder_Skewness |
|  |  |  | firstorder_TotalEnergy |
|  |  |  | firstorder_Uniformity |
|  |  |  | firstorder_Variance |
| GLCM features (n=22) | glcm_Autocorrelation | GLRLM features (n=16) | glrlm_GrayLevelNonUniformity |
|  | glcm_JointAverage |  | glrlm_GrayLevelNonUniformityNormalized |
|  | glcm_ClusterProminence |  | glrlm_GrayLevelVariance |
|  | glcm_ClusterShade |  | glrlm_HighGrayLevelRunEmphasis |
|  | glcm_ClusterTendency |  | glrlm_LongRunEmphasis |
|  | glcm_Contrast |  | glrlm_LongRunHighGrayLevelEmphasis |
|  | glcm_Correlation |  | glrlm_LongRunLowGrayLevelEmphasis |
|  | glcm_DifferenceAverage |  | glrlm_LowGrayLevelRunEmphasis |
|  | glcm_DifferenceEntropy |  | glrlm_RunEntropy |
|  | glcm_DifferenceVariance |  | glrlm_RunLengthNonUniformity |
|  | glcm_JointEnergy |  | glrlm_RunLengthNonUniformityNormalized |
|  | glcm_JointEntropy |  | glrlm_RunPercentage |
|  | glcm_Imc1 |  | glrlm_RunVariance |
|  | glcm_Imc2 |  | glrlm_ShortRunEmphasis |
|  | glcm_Idm |  | glrlm_ShortRunHighGrayLevelEmphasis |
|  | glcm_Idmn |  | glrlm_ShortRunLowGrayLevelEmphasis |
|  | glcm_Id |  |  |
|  | glcm_Idn |  |  |
|  | glcm_InverseVariance |  |  |
|  | glcm_MaximumProbability |  |  |
|  | glcm_SumEntropy |  |  |
|  | glcm_SumSquares |  |  |
| GLSZM features (n=16) | glszm_GrayLevelNonUniformity | GLDM features (n=14) | gldm_DependenceEntropy |
|  | glszm_GrayLevelNonUniformityNormalized |  | gldm_DependenceNonUniformity |
|  | glszm_GrayLevelVariance |  | gldm_DependenceNonUniformityNormalized |
|  | glszm_HighGrayLevelZoneEmphasis |  | gldm_DependenceVariance |
|  | glszm_LargeAreaEmphasis |  | gldm_GrayLevelNonUniformity |
|  | glszm_LargeAreaHighGrayLevelEmphasis |  | gldm_GrayLevelVariance |
|  | glszm_LargeAreaLowGrayLevelEmphasis |  | gldm_HighGrayLevelEmphasis |
|  | glszm_LowGrayLevelZoneEmphasis |  | gldm_LargeDependenceEmphasis |
|  | glszm_SizeZoneNonUniformity |  | gldm_LargeDependenceHighGrayLevelEmphasis |
|  | glszm_SizeZoneNonUniformityNormalized |  | gldm_LargeDependenceLowGrayLevelEmphasis |
|  | glszm_SmallAreaEmphasis |  | gldm_LowGrayLevelEmphasis |
|  | glszm_SmallAreaHighGrayLevelEmphasis |  | gldm_SmallDependenceEmphasis |
|  | glszm_SmallAreaLowGrayLevelEmphasis |  | gldm_SmallDependenceHighGrayLevelEmphasis |
|  | glszm_ZoneEntropy |  | gldm_SmallDependenceLowGrayLevelEmphasis |
|  | glszm_ZonePercentage |  |  |
|  | glszm_ZoneVariance |  |  |
| NGTDM features (n=5) | ngtdm_Busyness |  |  |
|  | ngtdm_Coarseness |  |  |
|  | ngtdm_Complexity |  |  |
|  | ngtdm_Contrast |  |  |
|  | ngtdm_Strength |  |  |
| Wavelets features (n=728) | wavelet_LH_* (n=182) | LoG features (n=273) | log_sigma_1_0_* (n=91) |
|  | wavelet_HL_* (n=182) |  | log_sigma_3_0_* (n=91) |
|  | wavelet_LL_* (n=182) |  | log_sigma_5_0_* (n=91) |
|  | wavelet_HH_* (n=182) |  |  |

GLCM = Gray-level co-occurrence matrices; GLRLM, Gray-level run length matrix; GLSZM, Gray-level size zone matrix; GLDM, Gray level dependence matrix; NGTDM, Neighborhood Gray Tone Difference Matrix; LoG, Laplacian of Gaussian.

**eTable 5**. Top 20 features by RFECV ranking

| Feature | Rank |
| --- | --- |
| wavelet-LHH_glszm_SmallAreaEmphasis | 1 |
| wavelet-HLH_glszm_SizeZoneNonUniformityNormalized | 1 |
| wavelet-LLH_firstorder_Kurtosis | 2 |
| wavelet-HLH_glszm_SmallAreaLowGrayLevelEmphasis | 3 |
| wavelet-HLH_glszm_SmallAreaEmphasis | 4 |
| wavelet-LHL_glcm_Correlation | 5 |
| wavelet-HLL_glcm_Correlation | 6 |
| wavelet-HHH_glcm_Imc1 | 7 |
| wavelet-HLH_gldm_SmallDependenceLowGrayLevelEmphasis | 8 |
| wavelet-HLL_glcm_Idmn | 9 |
| log-sigma-3-0-mm-3D_firstorder_Kurtosis | 10 |
| log-sigma-5-0-mm-3D_gldm_DependenceEntropy | 11 |
| wavelet-HHH_firstorder_Maximum | 12 |
| log-sigma-5-0-mm-3D_ngtdm_Busyness | 13 |
| original_glcm_Imc1 | 14 |
| wavelet-HHH_gldm_DependenceEntropy | 15 |
| wavelet-HLL_glszm_ZoneEntropy | 16 |
| wavelet-HLH_firstorder_Skewness | 17 |
| wavelet-HLL_glcm_Imc2 | 18 |
| wavelet-LLH_glcm_ClusterShade | 19 |

**eTable 6.** Radiomic features in different scanning protocols

| Features | Scanners | Median (IQR) | P-value |
| --- | --- | --- | --- |
| wavelet-LHH_glszm_SmallAreaEmphasis |  |  | 0.294 |
|  | GE Discovery MR750w | 0.310 (0.275 -0.359) |  |
|  | GE Discovery MR750 | 0.243 (0.196 - 0.327) |  |
|  | SIEMENS Skyra | 0.290 (0.258 - 0.342) |  |
|  | SIEMENS Avanto | 0.278 (0.262 - 0.282) |  |
|  | UIH uMR780 | 0.296 (0.287 - 0.304) |  |
|  | UIH uMR588 | 0.280 (0.259 - 0.377) |  |
| wavelet-HLH_glszm_SizeZoneNonUniformityNormalized |  |  | 0.141 |
|  | GE Discovery MR750w | 0.163 (0.147 - 0.191) |  |
|  | GE Discovery MR750 | 0.139 (0.130 - 0.187) |  |
|  | SIEMENS Skyra | 0.170 (0.146 - 0.196) |  |
|  | SIEMENS Avanto | 0.185 (0.153 - 0.206) |  |
|  | UIH uMR780 | 0.181 (0.154 - 0.208) |  |
|  | UIH uMR588 | 0.140 (0.130 - 0.144) |  |
